# Motor function, muscle strength and health-related quality of life of children perinatally infected with HIV

**DOI:** 10.4102/sajp.v78i1.1812

**Published:** 2022-11-30

**Authors:** Cassandra V. Rego, Joanne L. Potterton

**Affiliations:** 1 Department of Physiotherapy, Faculty of Health Sciences, University of the Witwatersrand, Johannesburg, South Africa

**Keywords:** human immunodeficiency virus (HIV), perinatal, gross motor function, muscle strength, health-related quality of life

## Abstract

**Background:**

Gross motor delays are common in infants and preschool children infected with human immunodeficiency virus (HIV). These delays persist in children of school-going age and may affect participation in classroom and playground activities; however, the extent of the problem is poorly understood in this age group.

**Objectives:**

Our study aimed to determine the motor function, muscle strength and health-related quality of life (HRQoL) in children aged 5–10 years who were perinatally infected with HIV.

**Methods:**

In our cross-sectional study, participants were recruited using convenience sampling from a Gauteng HIV clinic. Participants were assessed using the Movement Assessment Battery for Children, Second Edition (MABC-2), standing broad jump test (SBJT), Paediatric Quality of Life Inventory^TM^ (PedsQL) and a sociodemographic questionnaire.

**Results:**

Thirty children participated in our study. The MABC-2 showed 60% of the children assessed were either at risk of developmental delay or were already delayed, with the domain of manual dexterity being most affected. The SBJT showed female participants had weaker muscle strength than males. The mean total score on the PedsQL was 81%, with the subscales ranging from very high quality of life scores to moderately high quality of life scores, with emotional functioning having one of the lower overall scores.

**Conclusion:**

Children who have been perinatally infected with HIV are at significant risk of delayed motor function. Muscle strength is also an area of concern, as is emotional HRQoL. Further research and implementation of holistic rehabilitation programmes are needed.

**Clinical implications:**

Children with HIV need to be prioritised for developmental screening throughout childhood. Health promotion and early intervention need to be at the forefront of our fight against this pandemic.

## Introduction

Human immunodeficiency virus (HIV) is a constantly changing virus which is known to negatively impact infant and child development (Brassell & Potterton 2019; Melhuish & Lewthwaite 2018; Potterton, Hilburn & Strehlau 2016). In South Africa (SA), and according to the Joint United Nations Programme on HIV/AIDS (UNAIDS) 2020, there are 340 000 children living with HIV, only 47% of whom are receiving antiretroviral therapy (ART) (UNAIDS 2020). The vertical transmission rate in SA from mother to child, including breastfeeding, stands at 3.31% (UNAIDS 2020). The absolute CD4 counts, or the total amount of CD4 cells per mm^3^ of blood, is used to determine whether a child has immune suppression and its extent. The normal CD4 count for children between the age of 1 and 5 is 1000 cells/mm^3^ and between the ages of 6 and 12 is 500 cells/mm^3^ (Berhan 2011). As a result of rapid advances in medical management and care, quality of life and life expectancy in adults and children living with HIV have improved (Melhuish & Lewthwaite 2018). Despite these advances, children perinatally infected with HIV continue to face the lifelong challenges associated with living with a chronic disease (Comley-White, Potterton & Ntsiea 2022).

Health-related quality of life (HRQoL) has been investigated in a number of studies globally, which have found that children with HIV have a lower HRQoL, especially in relation to physical functioning, in comparison to noninfected control groups (Bomba et al. 2010; Melhuish & Lewthwaite 2018; Stevanovic, Tadic & Novakovic 2011). Children infected with HIV have a high risk of developing minimal to severe impairments in their development, which span all areas from visual, auditory, language, gross and fine motor to cognitive issues (Brassell & Potterton 2019). Van Rie et al. (2009) state that younger children infected with HIV are more likely to have severe motor and cognitive developmental delay than older children. In infants, global motor function has been shown to be the most significantly affected domain of development (Baillieu & Potterton 2008; Hilburn et al. 2011; Laughton et al. 2012; Potterton et al. 2016). Cognitive and language development may be moderately to severely affected (Baillieu & Potterton 2008; Potterton et al. 2009; Van Rie et al. 2009). Selective delays in executive function, visual-spatial tasks and processing speed issues have been identified (Van Rie et al. 2009).

Studies by Boyede et al. (2013), Lowick, Sawry and Meyers (2012) and Potterton et al. (2016) have investigated preschoolers affected by HIV. All three studies found that children in preschool have significant developmental delays, with gross motor development not necessarily the most adversely affected domain (Boyede et al. 2013; Lowick et al. 2012; Potterton et al. 2016). Of note, Boyede et al. (2013) state that socio-economic factors such as lower maternal age, lower levels of education and a low level of income correlate with a decrease in cognitive function in school-going children infected with HIV (Boyede et al. 2013). There are many reasons that motor function can be disrupted in a child who is HIV positive, including HIV encephalopathy and undiagnosed myopathy (Potterton et al. 2016).

Muscle weakness and myopathy are other known effects of HIV and may be because of the abnormal protein metabolism, malabsorption and depleted protein reserves (Potterton et al. 2021). However, if HIV is well controlled with early use of ART and lower viral loads, children infected with HIV have comparable muscle strength to children without HIV in the same age category (Potterton et al. 2021).

There is a need for further studies on preschoolers and young children infected with HIV and their motor function and quality of life. Our study therefore aimed to determine the motor function, muscle strength and the HRQoL of children aged 5–10 who were perinatally infected with HIV. It was hypothesised that our study would show that muscle strength, physical activity and motor function are decreased in children between the ages of 5 and 10 who were perinatally infected with HIV. This will help researchers and clinicians better understand the functional challenges school-aged children living with HIV may experience.

## Method

In this cross-sectional study, each child was assessed at a single time point during a routine clinic visit. Participants were drawn from an urban public HIV clinic which serves adults and children. The facility has a catchment area of approximately 1.2 million people and serves the surrounding urban areas and informal settlements.

Children were included if they met the following criteria: male or female children aged from 5–10 years, perinatally infected by HIV, HIV-positive status confirmed by PCR, child on ART and accompanied by a parent or legal guardian, and participants had to be ambulatory. Children were excluded if they were declared not medically fit for assessment by the medical officer at the clinic; this included children who had a fever or tachypnoea, as well as any congenital or musculoskeletal conditions not related to HIV that prevented them from participating in the assessments. Thirty participants were included to meet the central limit theorem (CLT) (Frost 2018). The CLT suggest a sample size of 30 participants is adequate, as the sample size is guided by the sample’s distribution across the population. The CLT states ‘given a sufficiently large sample size, the sampling distribution of the mean for a variable will approximate a normal distribution regardless of that variable’s distribution in the population’ (Frost 2018).

## Procedure

The first author and a nurse recruited participants who attended the clinic and met the inclusion criteria. They were invited to participate in our study and were provided with an information sheet, a consent form and an assent form. They were given 30 min to read the documentation and ask any questions. An interpreter was available if needed to make sure the participant and the parent or guardian had a clear understanding of what our study entailed. If participants and their parents or guardians agreed and signed both the consent form and the assent form, the assessments took place on the same day by an experienced paediatric physiotherapist employed at the facility. All children agreed verbally to participate in the presence of their parent or guardian, and children 7 years and older completed the assent process. The participants were first seen by the medical officer, and thereafter they came to the physiotherapy room. This room is within the clinic and is a comfortable and private environment. A participant code was assigned to all participants.

Firstly, a general self-constructed socio-economic and demographic questionnaire was administered to the parent or guardian as well as the participant. Following this, the Paediatric Quality of Life Inventory^TM^ (PedsQL) was administered by the first author, with the help of an interpreter, if needed. The PedsQL young child report for ages 5–7 and child report for ages 8–12 were used. The PedsQL 4.0 Generic Core Scale is the most widely used measure for child HRQoL (Amin et al. 2012) and has been shown to be valid in children with HIV as well as those who are uninfected (Banerjee, Pensi & Banerjee 2010).

Secondly, the collection of anthropometric data took place in the form of height and weight so that body mass index (BMI) could be calculated. Height and weight were measured with a calibrated Seko stadiometer electronic scale.

The standing broad jump test (SBJT) was then conducted. Several tests can evaluate lower body muscular strength. Of the available muscular strength tests, the SBJT correlates strongly with other tests of upper and lower muscular strength and is considered a reliable, practical, time-efficient, low-cost and low–participant burden measure that provides a general index of muscular strength in youth (Hardy et al. 2018). The SBJT is a valid, reliable and feasible field-based test which can evaluate explosive strength of the lower limbs and physical fitness (Thomas et al. 2020). This test was carried out on a hard surface, and a starting line was marked off on the floor with masking tape. The participants were barefoot and were asked to stand with their toes behind the starting line, keeping their feet slightly apart (Hardy et al. 2018). They were then told to bend their knees – the amount of flexion was the participant’s choice – and then their arms were placed behind them (Krishnan et al. 2017). They were then asked to jump as far as they could, using both feet at take-off and landing (Hardy et al. 2018) and swinging their arms forward as they jumped (Krishnan et al. 2017). Children were allowed two attempts. The distance from the start line to the back of their heel that was closest to the line was measured in centimetres (Hardy et al. 2018) using a tape measure. As a result of some language barriers experienced, a test attempt with a demonstration was given before their two final recorded attempts, to make sure that the participant understood what was expected. The SBJT has been used in studies in many countries around the world, including SA, and has been found to be both valid and reliable (Armstrong, Lambert & Lambert 2011; Krishnan et al. 2017; Monyeki et al. 2005).

Lastly, the participant undertook the Movement Assessment Battery for Children, Second Edition (MABC-2) test, which was performed according to their age group. This outcome measure encompassed eight tests under three subsections: balance, ball skills and manual dexterity. The test was administered according to the guidelines provided, and results were recorded on the test form. Information demonstrating how to administer the MABC-2 can be found in the manual written by Henderson, Sugden and Barnett (2007). The MABC-2 has been shown to be valid, reliable and a good identifier of motor deficits in children aged 3–16 years of age (Henderson et al. 2007; Smits-Engelsman et al. 2008). Currently, the MABC-2 has no norms for SA children; however, it has been used successfully in studies across various conditions (Corten & Morrow 2020; Van der Walt, Plastow & Unger 2020).

## Data management

Data were captured in a Microsoft Excel spreadsheet (Microsoft Corporation, Redmond, Washington, United States) and then imported into the Statistical Package for the Social Sciences (SPSS) version 28.0 (IBM Corporation, Armonk, New York, United States) for statistical analysis. Descriptive data were used to summarise the clinical and demographic data. Means and standard deviations or frequencies and percentages were used as appropriate. Appropriate measures of association were used to determine association between muscle strength and motor function and clinical and demographic factors. Pearson’s correlation was used to determine the association between variables. The Shapiro–Wilk test was used to test for normality. A *p*-value ≤ 0.05 was considered significant.

### Ethical considerations

Unconditional approval was obtained from the University of the Witwatersrand Human Research Ethics Committee (Medical) (reference number: M190656). The identified population group chosen were observed to be vulnerable; thus, all ethical guidelines from both the Health Professions Council of South Africa (HPCSA) and the Declaration of Helsinki were adhered to. The permission document, signed by participants, allowed for permission to access patient clinic files. All participants assessed in our study were referred to appropriate allied health or medical practitioners if results indicated a need.

## Results

Thirty children participated in our study. All data in our study were normally distributed.

The demographic and anthropometric data are presented in Table 1.

**TABLE 1:** Demographic and anthropometric results of participants (*n* = 30).

Factors of comparison	Minimum	Maximum	Mean	Standard deviation
Age (years)	5	10.05	7.76	1.66
Height (cm)	100.00	132.20	119.59	7.49
HAZ (cm)	−2.61	1.68	0.00	1.00
Weight (kg)	14.50	34.00	22.74	4.33
WAZ (kg)	−1.90	2.60	0.00	1.00
BMI	11.98	19.45	15.75	1.58
BMIZ	−2.39	2.35	0.00	1.00

HAZ, height-for-age *z*-score; WAZ, weight-for-age z-score; BMI, body mass index; BMIZ, body mass index *z*-score.

The mean age for participants was 7.73 years, with 13 female and 17 male children. Eleven children were in the 5–7-year age category, and 19 children were 8–10 years of age. The mean BMI was 15.75 kg/m^2^. On average, the children had a normal height and weight for age as well as a normal BMI, as evidenced by their height-for-age *z*-score (HAZ), weight-for-age z-score (WAZ) and body mass index *z*-score (BMIZ) scores (Table 1). All anthropometric data were compared with the World Health Organization (WHO) growth charts, developed from the WHO Multicentre Growth Reference Study (Garza & De Onis 2004). All participants in the sample were on ART.

Almost 75% (73.3%) of the parents or guardians reported that they received a social grant, whereas 26.7% reported that they did not receive any social grant. Furthermore, 70% of parents or guardians who reported receiving a social grant stated that they received a child support grant. The remaining 30% of the parents or guardians who received a grant did not specify which type of grant was received.

**FIGURE 1:**
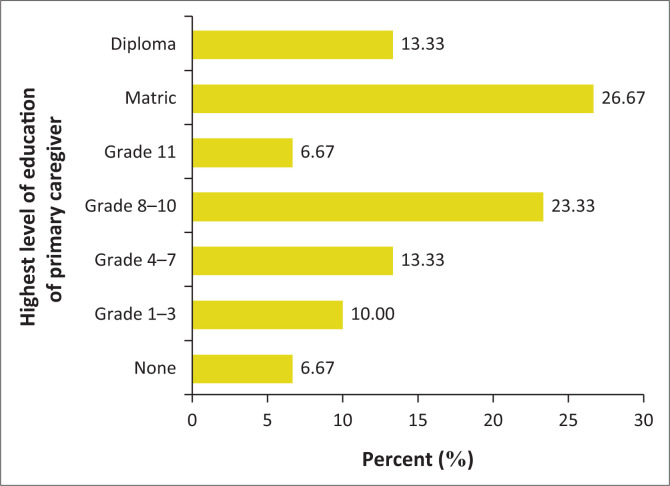
The primary parent or guardian’s highest level of education (*n* = 30).

The parent or guardian’s education levels are presented in Figure 1.

Eighty percent of the participants and their parents or guardians relied on public transport. This could be an inference of the socio-economic status of the families attending the clinic. Other socio-economic indicators showed that only 26.67% of the parents or guardians had a matric (Grade 12) certificate, 56.7% of the participants’ parents or guardians were unemployed and 73.3% of the parents or guardians received financial aid in the form of a government grant. Thirty percent of parents or guardians who were employed were working in unskilled jobs. Parents’ or guardians’ education has previously been found to have an association with developmental outcomes of children (Engle et al. 2011; Walker et al. 2011). The average age of parents or guardians was 40.59 years (SD 11.25).

The results of the MABC-2 are presented in Table 2.

**TABLE 2:** Individual components of the Movement Assessment Battery for Children - 2 scores classified into zones (traffic light system) (*n* = 30).

Factors of comparison	Green		Amber		Red	
	*n*	%	*n*	%	*n*	%
Manual dexterity	13	43.33	3	10	14	46.67
Aiming and catching	22	73.33	1	3.33	7	23.33
Balance	20	66.67	3	10	7	23.33
**Totals**	**12**	**40**	**7**	**23.3**	**11**	**36.7**

The results of the MABC-2 show that the aiming and catching and balance components had better performance by participants than the manual dexterity component. The manual dexterity component had the worst percentile score on average. Sixty percent of the participants fell into the amber or red zone, thus at high risk of delay or already delayed in their development of manual dexterity. The total MABC-2 scores placed almost a quarter (23.3%) of the sample in the amber zone, which indicated that there was a high risk of motor delay, and 36.7% of the sample fell into the red zone, which indicated that they definitely have motor delay; this means that only 40% of children presented with typical motor performance and fell in the green zone (Table 2). The mean total score of the MABC-2 was 60.43, which placed participants in the 21.18 percentile. This places participants on average in the green zone. All data were normally distributed.

The results from the SBJT are presented in Table 3.

**TABLE 3:** Standing broad jump test scores (*n* = 30).

Factors of comparison	Minimum	Maximum	Mean	Standard deviation	Skewness		Kurtosis	
					Statistic	Standard error	Statistic	Standard error
SBJT (cm)	63	145	99.00	24.62	−0.18	0.43	−1.23	0.83

SBJT, standing broad jump test.

The mean SBJT score was 99 cm, with the furthest length jumped being 145 cm. The mean length jumped by male participants was 107.64 cm, while for female participants it was 87.70 cm. This indicated that girls overall had weaker muscle strength than boys, with a *p*-value of 0.02, thus statistically significant.

The PedsQL (see Table 4) showed that school and emotional functioning were the lowest scoring areas for these participants. Fifty-nine per cent of the variance of quality of life is explained by emotional functioning. The PedsQL demonstrated an almost normal distribution. Only the subscale for social functioning was slightly negatively skewed; however, on all other subscales it was normally distributed. Both social functioning and school functioning are slightly platykurtic, indicating a more flattened distribution.

**TABLE 4:** Paediatric Quality of Life Inventory^TM^ results (*n* = 30).

Factors of comparison	Minimum	Maximum	Mean	Standard deviation
Physical functioning	62.50	100	87.50	11.10
Emotional functioning	30	100	77.33	21.65
Social functioning	30	100	80.67	17.41
School functioning	20	100	75.17	18.777
PedsQL total mean score	60.89	97.82	81.34	10.22

PedsQL, Paediatric Quality of Life Inventory^TM^.

The participants in our study on average had a viral load of 8920 copies/mL and CD4 count of 1064 cells/mm^3^.

## Correlations

The SBJT was positively and significantly related to manual dexterity of the MABC-2 (*r* [30] = 0.53; *p* < 0.003), as well as balance (*r* [30] = 0.45; *p* < 0.01) and total MABC-2 (*r* [30] = 0.52; *p* < 0.003). This indicated that the higher the participants’ SBJT score was, the higher their MABC-2 scores were as well. Furthermore, the proportion of variance explained by both manual dexterity and SBJT is *r*^2^ = 0.28, meaning the amount of variation that can be explained by both manual dexterity and SBJT, is 28%. The relationship between the SBJT and the MABC-2 was very strong, and all of the MABC-2’s components were moderate to strong and significantly related. This could be because of the fact that all motor functions require an adequate amount of muscle strength in order to complete them. The authors know from previous studies carried out (Humphries, Potterton & Mudzi 2014; Potterton et al. 2021) that muscle strength is greatly affected in children with HIV, unless their viral loads are adequately controlled. As the participants in our study were not all virally suppressed, the lower scores in the SBJT, muscle strength, could thus be one of the contributing factors as to why the MABC-2 showed so many of the participants to be delayed. This was found to be similar for the other components such as balance (*r*^2^ = 0.20) and total MABC-2 scores (*r*^2^ = 0.27). Overall, the SBJT had a significant positive relationship to the MABC-2 and all its components (see Table 5).

**TABLE 5:** Correlations between CD4 count, viral load and all outcome measures (*n* = 30).†

Factors of comparison	Tests	CD4 count (cells/mm^3^)	Viral load (copies/mL)	Manual dexterity	Aiming and catching	Balance	Total MABC-2	SBJT	PedsQL total
CD4 count (cells/mm^3^)	Pearson correlation	-	-	-	-	-	-	-	-
	*n*	22	-	-	-	-	-	-	-
Viral Load (copies/mL)	Pearson correlation	−0.05	-	-	-	-	-	-	-
	Sig. (2-tailed)	0.84	-	-	-	-	-	-	-
Manual dexterity	Pearson correlation	−0.11	−0.00	-	-	-	-	-	-
	Sig. (2-tailed)	0.63	0.99	-	-	-	-	-	-
Aiming and catching	Pearson correlation	−0.21	0.13	0.47**	-	-	-	-	-
	Sig. (2-tailed)	0.344	0.50	0.01	-	-	-	-	-
Balance	Pearson correlation	−0.11	−0.36	0.44*	0.26	-	-	-	-
	Sig. (2-tailed)	0.64	0.05	0.02	0.171	-	-	-	-
Total MABC-2	Pearson correlation	−0.106	−0.153	0.82**	0.57**	0.81**	-	-	-
	Sig. (2-tailed)	0.6438	0.42	0.00	0.00	0.00	-	-	-
SBJT	Pearson correlation	−0.30	−0.16	0.53**	0.34	0.46*	0.52**	-	-
	Sig. (2-tailed)	0.18	0.39	0.00	0.07	0.01	0.00	-	-
PedsQL total mean score	Pearson correlation	−0.11	−0.15	−0.23	−0.30	0.33	0.01	−0.03	-
	Sig. (2-tailed)	0.62	0.44	0.23	0.12	0.07	0.98	0.86	-

MABC-2, Movement Assessment Battery for Children, Second Edition; PedsQL, Paediatric Quality of Life InventoryTM; SBJT, standing broad jump test.

† , Only 22 participants’ viral load data were available.

* , Correlation is significant at the 0.05 level (2-tailed);

** , Correlation is significant at the 0.01 level (2-tailed).

## Discussion

Children living with HIV have been widely shown to be at risk of poor growth outcomes (Brassel & Potterton 2019). Similarly, another study carried out in Johannesburg, SA, on children perinatally infected with HIV showed that 12% of the participants were underweight and 16% of the participants were stunted for their age (Potterton et al. 2021).

This is notably different from the participants in our study, where very few of the participants were malnourished or stunted. A reason for this could be the rising inactivity of children with HIV. A study performed on adolescents with HIV in Brazil showed that there was a large number of adolescents who live sedentary lifestyles and were generally not involved in physical activity (Tanaka et al. 2015). Various other studies have also stated that the effects of HIV on protein metabolism, metabolic abnormalities such as lipodystrophy and poor nutrition lead to a more sedentary lifestyle (Humphries et al. 2014; Macdonald et al. 2017; Somarriba et al. 2013). Physical activity levels, diet and metabolism all need to be investigated in more detail in this population.

We showed that 36.7% of primary parents or guardians were employed, with the majority of them (30%) working in unskilled jobs. A large majority of the women worked as domestic workers, while the other 56.7% (17) of parents or guardians were unemployed. A Kenyan study on motor skill attainment and nutrition in children on ART reported a mere 5% of parents or guardians as unemployed, although many who were classified as employed had only casual jobs (Nkirote et al. 2019), while in our study, 56.7% of parents or guardians were unemployed. South Africa is arguably one of the wealthier countries in sub-Saharan Africa, so it is of interest that the unemployment rate of parents or guardians in our study’s sample is so much higher than the sample in the Kenyan study. Notably, much of the data were collected during the coronavirus disease 2019 (COVID-19) pandemic – a time when many people lost their jobs because of the lockdown restrictions.

Less than half (40%) of the parents or guardians had either matric (Grade 12) (26.67%) or some form of tertiary education (13.33%). This is similar to a study performed in SA by Potterton et al. (2009) that showed only 26.7% of the parents or guardians reached matric (Grade 12) (Potterton et al. 2009); thus, overall, the level of education across parents or guardians was generally low. This can affect motor outcomes, possibly because of the low level of exposure to educational toys, books and multimedia in areas struggling with severe poverty and low maternal literacy (Van Rie et al. 2009). Maternal education has been shown to be an important factor associated with child development (Engle et al. 2011; Walker et al. 2011), and so efforts to improve child outcomes should include programmes for their mothers and parents or guardians as well.

Various studies have measured motor development and performance in children living with HIV, some using the MABC-2 and others using tests such as the Griffiths Mental Development Scales (GMDS) and the Bayley Scales of Infant Development (Baillieu & Potterton 2008; Lowick et al. 2012; Sherr et al. 2014; Van Rie et al. 2009). The results of the MABC-2 clearly demonstrate that 60% of the participants are deemed at risk or definitely delayed across their gross motor functions. Only 40% of the participants managed to score in the green zone, above the 15th percentile. Overall, participants either excelled with good scores or they performed poorly. Very few participants fell into the amber zone, even when analysing each component separately. These findings are similar to those reported by Benjamin-Damons (2019), who found that 71% of the children living with HIV fell into an at-risk category, that is, either into the red or amber zones (Benjamin-Damons 2019). According to the MABC-2 results, manual dexterity was the most severely affected domain. These results are similar to those reported by Benjamin-Damons (2019). Neither of the populations had been previously screened or assessed; thus, these challenges could persist and negatively impact participation and school performance.

A study which investigated development in preschool children living with HIV and used the Griffiths Scales of Mental Development (GSMD) showed that 26% of their sample struggled with hand–eye coordination and/or fine motor control (Potterton et al. 2016). Thus, this is an area where more research needs to be conducted, as this is a vital set of skills for daily functioning and school tasks.

Compared with the norms developed across Europe (Thomas et al. 2020), there were eight female participants whose jump length was below the 10th percentile. That means that 61.5% of the female participants had a short jump length. None of the male participants scored below the 10th percentile. The normative data only started from age 6 (Thomas et al. 2020); thus, the 45-year-old men could not be compared with the normative data set. According to the European study, the boys are able to jump further than girls, which is comparable to the results of our study. A study from Johannesburg, SA, gave one reason that girls were found to be more physically inactive and participate in more sedentary lifestyles than boys at the same age (Wong et al. 2016). This would negatively impact the muscle strength of girls; thus, it gives rise to lower distance jump scores in the SBJT. A study was performed in SA on primary school children from multiple provinces between the ages of 6 and 13 years. They found that as children grow older, their jump length increases, as well as the fact that boys managed to jump a further distance than girls (Armstrong et al. 2011). The results of our study were also much lower than those of the Armstrong et al. (2011) study for both boys and girls, with their mean score for boys aged 6 being 117.1 cm (Armstrong et al. 2011).

The participants did not all have optimally controlled viral loads, as only 11 participants had viral loads that were undetectable. This could explain why our study’s participants had low scores on the SBJT in comparison to the European and SA norms (Armstrong et al. 2011; Thomas et al. 2020). Adherence to medication was not recorded, and it may have been a contributing factor, especially as data were collected during the COVID-19 pandemic when adherence was known to be a challenge.

Another reason could be that SA falls in the category of an upper-middle-income country according to the World Bank for 2021. However, the unemployment rate is 32.5% (Statistics South Africa 2020), and thus many SA people fall below the bread line, thus having limited funds. It is known that in order to build adequate muscle, especially when one has HIV as well, one would require adequate nutrition, especially protein (Humphries et al. 2014). In communities where there is a lack of adequate nutritious food, children are unable to maintain their nutrient stores and build muscle. Children on ART containing protease inhibitors have more central adipose tissue and may be slightly shorter and be stunted by 0.7 cm (Humphries et al. 2014). Children who are not on ART have a weaker immune system, and a correlation between CD4 counts and muscle strength has been found. However, once the child is on ART and the viral load starts to be controlled, the CD4 count rises, but their muscle strength does not concurrently increase; thus, the use of ART does not facilitate any increased muscle building (Humphries et al. 2014), and further strengthening may be needed. As a result of the socio-economic status of many of the families, this may not always be possible, thus also affecting the distance jumped scores overall.

The PedsQL was used to investigate the HRQoL of the children. The mean total score on the PedsQL was 81%, with a standard deviation of 18. This is deemed very high, as the subscales ranged from very high quality of life scores to moderately high quality of life scores. The results demonstrate that the largest variance in HRQoL was the emotional subscale. A study by Banerjee et al. (2010) found that children with HIV had lower HRQoL on the PedsQL in school, emotional and physical subscales. The results by Banerjee et al. (2010) are comparable with the lower scores in school and emotional subscales; however, the sample had an overall good HRQoL in the physical subscale. Another study performed on Thai children with HIV yielded very similar results to our study, with the lowest and second lowest subscales being school and emotional, respectively (Punpanich et al. 2010).

Furthermore, a study performed on children living with HIV and sensory neuropathy in SA had a total PedsQL score of 79.6%. The study by Benjamin-Damons (2019) used the scoring guidelines of the PedsQL, noting that > 50% was considered a good HRQoL score (Benjamin-Damons 2019). Interestingly, both our study and the study performed by Benjamin-Damons (2019) had very good HRQoL for the physical component of the PedsQL, despite the difficulties these children showed when assessed on a standard score for motor function and muscle strength. Thus, in relation to the given studies, overall, the participants in our study had a good HRQoL across all aspects tested.

Viral load had a negative and nonsignificant or very weak relationship with the MABC-2, SBJT and the PedsQL. Thus, the higher the viral load, the lower the scores on the respective outcome measures. Although this relationship is weak, possibly because of the small sample size, higher viral loads are associated with HIV encephalopathy (Donald et al. 2014; Mann et al. 2017) and muscle weakness (Humphries et al. 2014). Thus, we would expect the overall scores on the participants’ assessments across both motor function and muscle strength to be lower than those of a child who has well-controlled HIV.

There was a strong and significant relationship between BMI and balance (*r* = 0.57; *p* < 0.00), with BMI accounting for 33% of the variance in balance. Total motor functions are also significantly and positively correlated with BMI (*r* = 0.43; *p* < 0.02). Aiming and catching and manual dexterity were not significantly related to BMI. Previous research demonstrates different outcomes to our study with regard to its relationship with motor function; thus, more research is needed in this area for clarity. None of the participants in our study were found to be obese, which is of importance.

The SBJT was positively and significantly related to the MABC-2 components of manual dexterity (*r* [30] = 0.53; *p* < 0.00) as well as balance (*r* [30] = 0.45; *p* < 0.01) and the total MABC-2 score (*r* [30] = 0.52; *p* < 0.00). This indicates that the further the distance jumped in the SBJT, the higher their MABC-2 scores are as well. The relationship between SBJT and the MABC-2 was very strong, and all of the MABC-2’s components were moderate to strong and significantly related. This could be because of the fact that all motor functions require an adequate amount of muscle strength in order to complete them. We know from studies (Humphries et al. 2014; Potterton et al. 2021) that muscle strength is greatly affected in children with HIV, unless their viral loads are adequately controlled. As our participants were not all virally suppressed, the lower scores in the SBJT, muscle strength, could thus be one of the contributing factors as to why the MABC-2 showed so many of the participants to be delayed.

### Limitations

Our study was conducted during the unforeseen COVID-19 pandemic and subsequent lockdown, which affected its sample size. Despite the fact that all the data were normally distributed, a larger sample size would have been preferred in order for the results to be more broadly generalisable. As a result of the pandemic, collection of data from an HIV-uninfected and unexposed group was unattainable, and the addition of this comparator group would have strengthened our study.

## Conclusion

The ever-growing body of research clearly shows that with improved access to ART, children with HIV are able to live relatively healthy lives, albeit with a chronic health condition.

More than half the children in our study were found to be at risk of or already developmentally delayed in terms of motor function, with manual dexterity being the most severely affected domain. Female participants had lower SBJT scores, and thus poorer muscle strength in comparison to their male counterparts, although the authors note that it would be important to compare with an HIV-unexposed uninfected group of age and gender matched controls in future studies. Emotional HRQoL was the worst affected domain, but overall, the participants had a good score for HRQoL.

The need for developmental screening and monitoring is vital for this vulnerable population so that any disabilities or difficulties can be identified and intervention commenced. Physiotherapists need to be more involved at all levels of healthcare in a preventive and health promotion role. This could help to ensure that children living with HIV are referred for appropriate screening, and intervention for motor difficulties could be performed more timeously.
